# Improving Automatic Coronary Stenosis Classification Using a Hybrid Metaheuristic with Diversity Control

**DOI:** 10.3390/diagnostics14212372

**Published:** 2024-10-24

**Authors:** Miguel-Angel Gil-Rios, Ivan Cruz-Aceves, Arturo Hernandez-Aguirre, Martha-Alicia Hernandez-Gonzalez, Sergio-Eduardo Solorio-Meza

**Affiliations:** 1 Universidad Área Académica de Tecnologías de la Información, Universidad Tecnológica de León, Blvd. Universidad Tecnológica 225, Col. San Carlos, León 37670, Mexico; mgil@utleon.edu.mx; 2 CONAHCYT, Consejo Nacional de Humanidades, Ciencia y Tecnología (CONAHCYT), Centro de Investigación en Matemáticas (CIMAT), A.C., Jalisco S/N, Col. Valenciana, Guanajuato 36000, Mexico; 3 Centro de Investigación en Matemáticas (CIMAT), A.C., Jalisco S/N, Col. Valenciana, Guanajuato 36000, Mexico; artha@cimat.mx; 4 Unidad Médica de Alta Especialidad (UMAE), Hospital de Especialidades No.1. Centro Médico Nacional del Bajio, Instituto Mexicano del Seguro Social (IMSS), Blvd. Adolfo López Mateos S/N, León 37150, Mexico; martha.hernandez@imss.gob.mx; 5 División de Ciencias e Ingenierías, Universidad de Guanajuato, Campus León, Loma del Bosque 103, Col. Lomas del Campestre, León 37150, Mexico; se.solorio@ugto.mx

**Keywords:** coronary angiography, evolutionary algorithm, feature selection, population diversity, stenosis classification

## Abstract

This study proposes a novel Hybrid Metaheuristic with explicit diversity control, aimed at finding an optimal feature subset by thoroughly exploring the search space to prevent premature convergence. **Background/Objectives**: Unlike traditional evolutionary computing techniques, which only consider the best individuals in a population, the proposed strategy also considers the worst individuals under certain conditions. In consequence, feature selection frequencies tend to be more uniform, decreasing the probability of premature convergent results and local-optima solutions. **Methods**: An image database containing 608 images, evenly balanced between positive and negative coronary stenosis cases, was used for experiments. A total of 473 features, including intensity, texture, and morphological types, were extracted from the image bank. A Support Vector Machine was employed to classify positive and negative stenosis cases, with Accuracy and the Jaccard Coefficient used as performance metrics. **Results**: The proposed strategy achieved a classification rate of 0.92 for Accuracy and 0.85 for the Jaccard Coefficient, obtaining a subset of 16 features, which represents a discrimination rate of 0.97 from the 473 initial features. **Conclusions**: The Hybrid Metaheuristic with explicit diversity control improved the classification performance of coronary stenosis cases compared to previous literature. Based on the achieved results, the identified feature subset demonstrates potential for use in clinical practice, particularly in decision-support information systems.

## 1. Introduction

**Figure 1:**
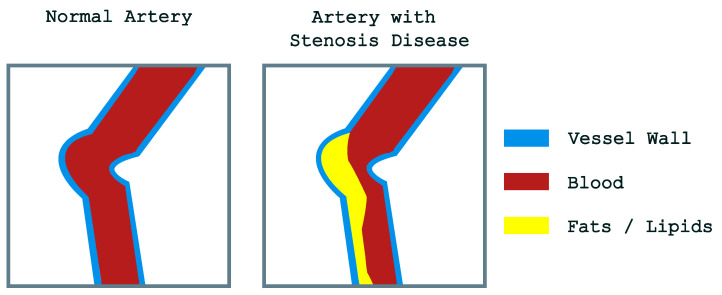
Schematic representation of a normal artery (**left**) and an obstructed artery caused by the accumulation of fats/lipids (**right**).

Coronary Artery Disease (CAD) is a leading cause of mortality worldwide. Coronary stenosis occurs due to the accumulation of harmful substances, such as fats and lipids, within the arteries, leading to an obstruction that disrupts normal blood flow. This condition can result in heart attacks, which often have fatal outcomes. Figure 1 illustrates a schematic representation of a normal artery (left) and an artery obstructed by the accumulation of fats and lipids (right).

**Figure 2:**
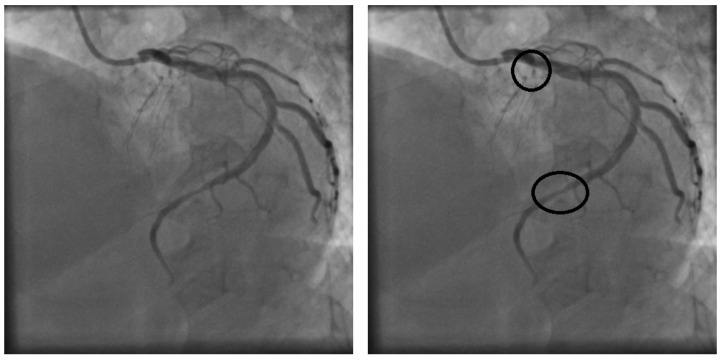
A coronary angiography (**left**) with corresponding stenosis areas labeled by the cardiology specialist (**right**).

Accurate diagnosis of coronary stenosis is crucial for quality of life and life expectancy [1]. The most widely used method for coronary stenosis detection is the visual examination of coronary angiography, which is produced using X-rays and a contrast medium that is injected into the patient [2]. The resulting X-ray spectrum can be printed on physical media or presented as a digital image or video. Cardiology specialists conduct the stenosis detection process by performing a thorough visual examination of the entire angiography. Based on their expertise, they identify and label areas with possible stenosis cases. Figure 2 shows a digital image of a coronary angiography with labeled stenosis cases by the specialist.

In the literature, the coronary stenosis problem has been addressed using various approaches, including vessel enhancement and segmentation methods, as well as machine learning and deep learning classification techniques. For instance, the use of spatial filters based on Gaussian kernels and the Hessian matrix were useful to enhance the main artery tree in order to extract certain features to identify specific stenosis cases [3,4,5,6].

Deep learning techniques, such as Convolutional Neural Networks, have also been applied to classify coronary stenosis [7,8]. However, these methods require large datasets to achieve a high classification performance in terms of accuracy [9,10]. Due to ethical and legal restrictions, medical image banks of coronary arteries with proper stenosis cases labeled by specialists are scarce, posing challenges to achieving optimal performance using deep learning techniques.

Automatic classification of coronary stenosis is challenging, as identifying relevant image features that distinguish between positive and negative stenosis cases is difficult. Moreover, low contrast and high noise levels complicate the detection of artery structures. In this context, digital image processing techniques aimed at vessel enhancement are vital, as different filtering and segmentation methods yield varied responses. For instance, spatial filters can enhance artery structures, enabling the extraction of morphological features. In addition, intensity and texture-related features can also be extracted [11]. This feature extraction is important for maximizing information retrieval from coronary angiographies [12]. However, when dealing with large feature sets, it becomes difficult to identify which features are most relevant for accurate classification [13].

Identifying an optimal subset of features that enhance classification performance is complex, as the computational cost of exploring all possible combinations is denoted by $O(2^{n})$, where *n* represents the number of involved features. In addition, computational search techniques require an objective function to evaluate solution performance. By considering these principles, automatic feature selection for coronary stenosis classification is expressed as a multi-objective function where the best solution is achieved by minimizing the number of selected features and maximizing the classification performance rate.

The automatic feature selection problem has been widely studied in the literature using distinct computational techniques such as population-based methods, evolutionary computing, and others [14,15,16]. A common issue with automatic search techniques is premature convergence, which prevents proper exploration of the search space and may overlook better solutions [17].

In the present work, a novel method for automatic feature selection in high-dimensional spaces is proposed. The method involves a hybrid metaheuristic with explicit diversity control used to generate new populations of potential solutions. The achieved results with their corresponding analysis, demonstrate the importance of this diversity control strategy in improving the classification performance for coronary stenosis by selecting a better suitable feature subset than traditional approaches reported in the literature. The key contributions of this study include the following:

- Identification of 16 features that are relevant for the classification of positive and negative coronary stenosis cases.
- Development of a feature vector consisting of 473 distinct features extracted from the original images and responses from vessel enhancement filters.
- Implementation of a diversity control strategy to decrease the probability of premature convergence during the automatic feature selection process.

The remainder of this paper is structured as follows: Section 2 discusses the theoretical concepts and methods underlying the proposed method. Section 3 provides a detailed description of the proposed method. Section 4 outlines the image database, consisting of positive and negative stenosis cases used for experimentation, and presents the results, comparing them with existing approaches. Finally, Section 5 discusses the conclusions and relevant findings of this study.

## 2. Methods

Working with feature datasets for classification tasks, generally involves feature extraction and feature selection stages. The feature selection task is difficult to carry out manually when the number of features is high. In consequence, addressing the problem with the use of search metaheuristics is adequate. The search metaheuristics are used to find a suitable feature subset, able to achieve optimal classification rates.

### 2.1. Feature Extraction

The feature extraction process is commonly one of the first stages in order to form a working dataset [18]. In the digital image processing area, several feature types are possible to be extracted such as intensity, texture and morphology. The intensity-based features are the most simple to extract since they can be computed directly from the original image. Based on each individual pixel intensity, there is possible to compute the minimum, maximum, median, average, variance and standard deviation measures. Texture-based features are also possible to be extracted from the original image. Almost all texture features are required to compute the Gray Level Co-Occurrence Matrix (GLCM) as a previous step [19]. Consequently, using the GLCM allows us to extract 14 distinct texture features using the Haralik Equations [11]. Additionally, morphological features provide valuable information about the shape, area and geometry of interest objects [14]. However, in order to obtain accurate measurements related to morphological features, the objects of interest must be enhanced from the original image. The use of filters in spatial and frequency domains plus other techniques from the literature, are commonly used to address the problem [20,21]. For instance, a linear multi-scale method is useful to enhance arterial segments that are able to be approximated to straight lines at distinct sizes and orientations [22]. In addition, the use of Gaussian-based filters are helpful to enhance curved artery structures using distinct curve amplitude scales [23,24]. With the arteries segmented from the rest of the image, it is possible to extract several morphological features such as the compactness, circularity ratio, elongatedness, and others [17].

Compactness is defined as the ratio of the square of the perimeter to the area of an artery segment. It is insensitive to uniform scale changes and can be computed as follows:

(1) $$C=\frac{P^{2}}{A},$$

where *C* represents the compactness, *P* is the perimeter of the artery segment and *A* is its corresponding area, measured in pixels.

**Figure 3:**
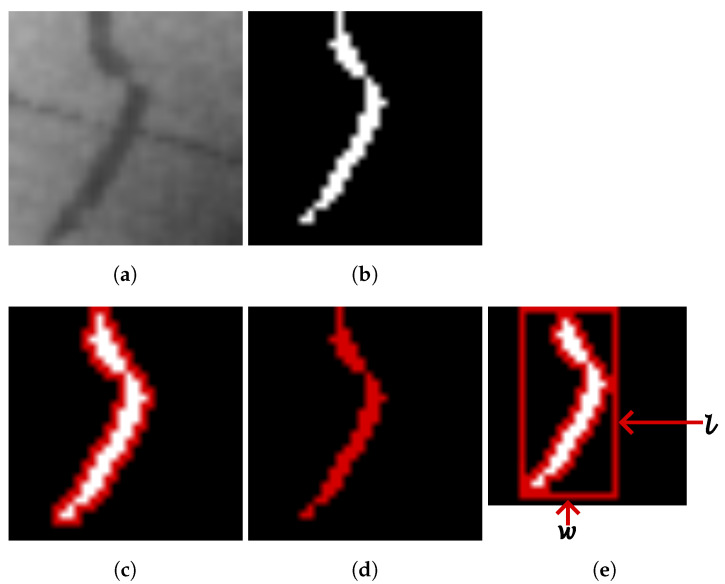
Original angiography patch with its corresponding vessel segmentation, highlighting three morphological features: perimeter, area and elongatedness. (**a**) Original image. (**b**) Artery segmentation. (**c**) Perimeter. (**d**) Area. (**e**) Elongatedness.

The elongatedness of a region is the ratio between the length *l*, and the width *w* of the minimum rectangle of the corresponding region. In Figure 3, an original angiography patch is shown with its corresponding vessel segmentation and three distinct morphological features: perimeter, area, and elongatedness.

In addition, the circularity ratio is a morphological feature that measures the ratio of the artery area to the area of a circle having the same perimeter. It can be computed as follows:

(2) $$R_{c}=\frac{4\pi A}{P^{2}},$$

where $R_{c}$ is the circularity ratio, *A* is the artery area and *P* is its corresponding perimeter.

On the other hand, the morphological Top-Hat operator was proposed by Eiho and Qian as a method for detection of artery trees [25]. The Top-Hat operator involves the operations of dilation and erosion. The dilation step is focused in the enhancement of the arteries by detecting local intensity variations by the use of a structure element. The structure element is a relevant component in the TopHat method because it defines the size and shape of the interest objects. For artery enhancement, the size of the structure element must be established based on the vessel width. In the erode step, the resultant noise is discriminated and the artery information is enlarged in order to obtain the enhanced image which content is then segmented into vessel-pixels and non-vessel pixels by applying a manual or automatic threshold.

### 2.2. Feature Selection

**Figure 4:**
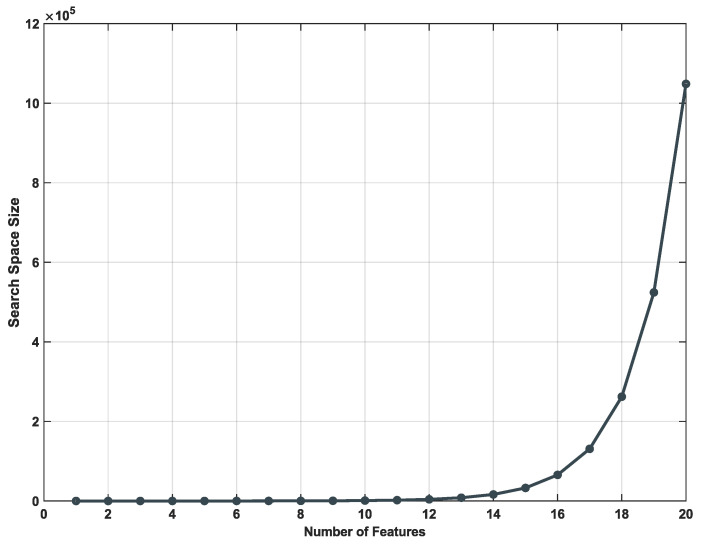
Search space size involving a feature set with size in the range [0,20].

Extracting the highest number of features to form a dataset, that will be used later for classification, is desirable [26]. However, identifying the relevance of each individual feature or combined with others often turns into a complicated task to be done analytically by hand. The number of distinct combinations involving all the extracted features is $O(2^{n})$, where *n* is the number of features. By considering all the possible combinations related with the number of involved features, the search space increments exponentially making difficult to work manually with them. Figure 4 illustrates the high-dimensional complexity increment considering a sample number of features in the range [1,20].

Consequently, the feature selection problem has been addressed with different approaches [27,28]. Because of the complexity involved in the feature extraction process, automatic search techniques have been applied to solve the problem. The use of population-based estimation of distribution algorithms has been relevant when working with coronary arteries [13,17].

### 2.3. Metaheuristics

Metaheuristics is a set of algorithms for solving high-dimensional optimization problems in discrete or continuous domain, where evolutionary algorithms is a representative subset. The evolutionary algorithms try to imitate the nature of population species in which, more adapted individuals have the highest possibility to create a new population, also known as the new generation, by using distinct operators. This behavior was adapted by John Holland in 1984 to create a computational method able to search for relevant solutions to complex problems that are difficult to be solved by hand or traditional analytical methods [29]. Subsequently, new approaches and techniques were proposed based on the Holland principles in order to generate new individuals that will conform the new generations.

#### 2.3.1. Boltzmann-Univariate Marginal Distribution Algorithm

The Boltzmann-Univariate Marginal Distribution Algorithm (BUMDA) is a variation of the original Univariate Marginal Distribution Algorithm. In evolutionary computation, an individual represents a possible solution to a given problem. In the feature selection problem, each individual is represented as a feature vector of size *n*, where *n* is the number of involved features. By considering this principle, each element inside the vector has a discrete value within the range [0,1], where 0 means that a specific feature is not selected and 1 means that the feature is selected in the corresponding generated solution.

Traditional evolutionary methods, such as the Genetic Algorithm (GA) [30], produce new solutions by taking two of the best individuals (called “parents”) from the current population at random. These parents are then split into two or more parts and, by mixing the parts of both parents, new individuals are created.

On the other hand, the population distribution estimation-based methods generate new solutions based on the distribution and frequency of each element (feature) by considering a rate of the population that are part of the current generation [31]. A variation of the original UMDA method was proposed by Valdez et al. in which, the individuals for the new generation are produced using the Boltzmann distribution as follows [32]:

(3) $$\mu =\sum_{j}W\left(X_{j}\right)x_{j},\;\mathrm{where}\;W\left(X_{j}\right)=\frac{g(X_{j})}{\sum_{X_{j}}g\left(X_{j}\right)},$$

(4) $$\nu =\sum_{j}W^{\prime }\left(X_{j}\right){\left(X_{j}-\mu \right)}^{2},\;\mathrm{where}\;W^{\prime }\left(X_{j}\right)=\frac{g(X_{j})}{\sum_{X_{j}}g\left(X_{j}\right)+1},$$

where μ is the fitness average, ν represents the fitness variance computed from all individuals in the population. $g(X_{j})$ is the fitness value corresponding to the $j^{th}$ individual in the population *X*. Consequently, taking a fraction of the current population, new individuals are produced as following:

(5) $$\theta^{t+1}=\left\{\begin{array}{cc} f(x_{\mathrm{npop}}) & \mathrm{if}\;t=1,\\ f(x_{\frac{\mathrm{npop}}{2}}) & \mathrm{if}\;f\left(x_{\frac{\mathrm{npop}}{2}}\right)>=\theta^{t},\\ f(x_{i}) & \mathrm{when}\;f\left(x_{i}\right)>=\theta^{t}{|}_{i=\frac{\mathrm{npop}}{2}+1}^{\mathrm{npop}}, \end{array}\right.$$

where $\theta^{t+1}$ represents the new population, $x_{npop}$ denotes all the individuals in the current population and, npop is the population size.

The concept of *fitness* or *objective function* measures the performance of each specific solution found by the metaheuristics. In automatic feature selection, the fitness measure of an individual can be computed evaluating its selected features by training a classifier and measuring the achieved accuracy.

The main advantage of BUMDA over other strategies is that it is governed by only three parameters: the number of generations, the population size, and the population selection rate. In addition, because of the Boltzmann distribution, the method increases the probability of generating diverse populations, thereby decreasing the risk of convergence to local-optima solutions.

#### 2.3.2. Simulated Annealing

The Simulated Annealing (SA) method is a metaheuristic technique used for the search of solutions in computation. The method starts with an initial solution that is refined into an iterative process in which, worst solutions are discriminated more strictly as the iterations progress increases. The SA method was abstracted from metallurgy and adapted as a computational automatic search strategy [33]. It is governed by three parameters: the initial temperature value, the temperature-changing step, and the final temperature. Throughout the iterative process, the temperature is decreased from the initial value to the final value by a constant step, as follows:

(6) $$P\left(\Delta E,T\right)=\frac{f\left(s^{\prime }\right)-f\left(s\right)}{T},$$

where, P(ΔE,T) is the probability computed from the Boltzmann distribution. $f(s^{\prime })$ represents a fitness value computed from an objective function in which the new solution is evaluated. f(s) denotes the fitness value obtained by evaluating the current solution in the objective function. *T* is the current temperature.

The SA is a single-solution technique. The method starts with an initial solution generated randomly or by some other manner. Until the iterative SA loop, the solution is altered and evaluated. The solution alteration is made by using the probability which comes from the Boltzmann distribution. This method tends to achieve better results when combined with some other strategy [17].

### 2.4. Support Vector Machine

The Support Vector Machine (SVM) is a classification method that uses the concepts of plane and hyperplane in order to perform a binary classification. It is made by projecting the original dimensional space to higher orders in which the classification can be performed adequately in terms of the accuracy metric [34,35]. The main advantage of the SVM is the efficiency in classification tasks where the data belonging to each class, overlaps considerably and, there is not spatial difference between the elements of each class. In order to generate higher dimensional orders, the SVM needs to find the data-points located at the boundaries of both classes. These data points are used as the support vectors to project the plane or hyperplane into a higher-dimensional order space as follows [36]:

(7) $$f\left(x\right)=W^{T}\phi \left(X\right)+b,$$

where *W* is the weight vector and normal to hyperplane, ϕ is the projection function or kernel, *b* is the bias or threshold and *X* is the data point to be classified.

## 3. Proposed Method Using a Diversity Strategy

The proposed method aims to improve the feature selection stage by implementing a population diversity strategy within the hybrid metaheuristic [37]. Initially, 473 distinct features were extracted from an image bank consisting of 608 patches of coronary angiographies. These feature sets include intensity, texture, and morphological feature types [38].

The automatic selection task becomes complicated in datasets with a large number of features. Even though search metaheuristics probe their effectiveness when applied to a 473-sized feature vector, fast convergence could limit the diversity of new solutions. This limitation decreases the probability of evaluating other suitable feature subsets that could yield higher coronary stenosis classification rates.

In order to decrease the probability of generating close-spatial solutions, which leads to an early convergence, the proposed method implements a diversity generation strategy.

The traditional search metaheuristics starts by generating a random number of solutions as the initial population. Then, each individual in the population is evaluated although a fitness function and ranked based on its performance measure. Subsequently, the individuals of the population are sorted based on its performance from the best to the worst. Next, the best individuals are selected using a *selection rate* parameter to create new individuals (solutions) until a finite number of generations are reached.

However, in high-complexity search spaces, using only the best individuals to produce new populations, it is possible to achieve an early biased solution [39]. Alternatively, by considering the worst individuals under controlled conditions, the population diversity is increased [37].

**Figure 5:**
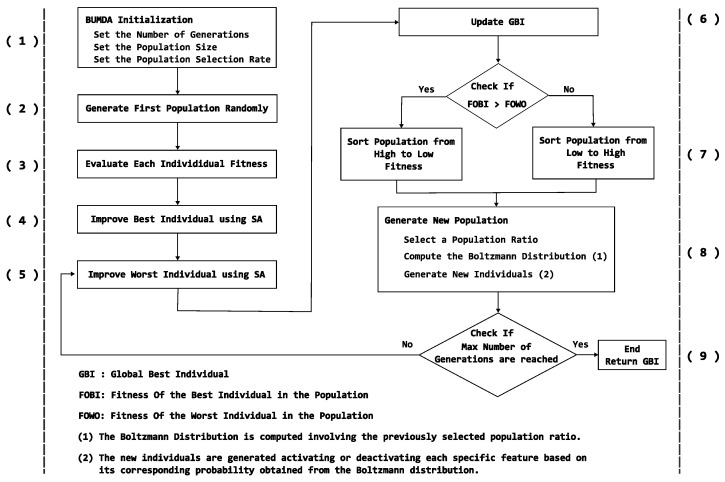
Overall process of the hybrid metaheuristic based on BUMDA and SA with diversity control, for automatic feature selection. Steps are as following. 1. BUMDA initialization. 2. First population generation. 3. Fitness computation for each individual in the current generation. 4. Improvement of the best individual in the current generation. 5. Improvement of the worst individual in the current generation. 6. Update of the global-best solution. 7. Sorting of the new population from the best to the worst individuals or viceversa. 8. Generation of the individuals for the new population based on the previous sorting. 9. Condition to end the process or continue until the maximum number of generations is reached.

Figure 5 illustrates the steps of the proposed method, which combines a hybrid metaheuristic with a population diversity strategy for automatic feature selection. The hybrid metaheuristic integrates the BUMDA and the SA techniques.

According to Figure 5, the proposed method starts by establishing the maximum number of generations, the population size and selection rate. Then, in step (2), the individuals of the population corresponding to the first generation, are randomly initialized. As described previously, each individual is a vector of discrete elements which can have a value of 0 or 1. The vector size corresponds to the number of involved features where, a value of 1 in some vector element means that the corresponding feature is selected and vice versa.

In step (3), each individual is evaluated. The fitness value is obtained by training a supervised classify-based model using only the selected features. In this case, a linear SVM was trained and tested using a holdout subset of instances, separated previously from the dataset. Subsequently, the classification performance is evaluated with the Accuracy metric, which is computed as follows:

(8) $$Accuracy=\frac{TP+TN}{TP+TN+FP+FN},$$

where TP, TN, FP and FN, corresponds to the True-Positive, True-Negative, False-Positive and False-Negative fractions, respectively.

In addition, the number of involved features are also considered applying a feature decreasing rate metric which is computed as follows:

(9) $$FDR=1-\frac{Number\;of\;Selected\;Features}{N},$$

where, FDR is the feature decreasing rate value and, *N* is the total number of involved features.

By considering Equation (9), when the number of selected features decrease, the feature decreasing rate is incremented. By involving the FDR along with the classification accuracy, the final fitness value for each specific individual can be obtained by maximizing the objective function.

In steps (4) and (5), is selected the worst individual along with the best one from the current BUMDA generation. Then, the two individuals are refined by applying the SA method. Subsequently, in the step (6), the global best individual ($G_{Best}$) is updated only if some of the improved individuals have a higher fitness value than the current $G_{Best}$.

Following the process, in step (7), the strategy to keep diversity of individuals in the population is relevant. If the best SA-refined individual was the worst in the BUMDA current population, the population is sorted from the worst to the best. In other case, the population is sorted in the traditional way: best individuals will occupy the first places. By applying this strategy, in step (8), some new populations will be generated by considering the worst individuals from the current one. Otherwise, the best individuals will be considered to produce new generations. The method ends when the maximum number of generations is reached.

Finally, in order to asses the achieved classification performance, the Jaccard coefficient, F1-score, Sensitivity and Specificity metrics were additionally implemented, which can be computed using the TP, TN, FP and FN values. The Jaccard coefficient ($J_{c}$) is computed as follows:

(10) $$J_{c}=\frac{TP+TN}{\left(A+P)-(TP+TN\right)},$$

where, *A* is the number of actual values and *P* is the number of predicted values.

The use of the $J_{c}$ metric is relevant to asses the classification performance accuracy because the $J_{c}$ is focused in the measurement of correctly classified instances and, incorrect classification cases have a higher penalization rate than in the Accuracy metric.

The F1-score ($F_{1}$) metric is computed as follows:

(11) $$F_{1}=\frac{2TP}{2TP+FP+FN}$$

The Sensitivity metric is computed as follows:

(12) $$Sensitivity=\frac{TP}{TP+FN}$$

The Specificity metric is computed as follows:

(13) $$Specificity=\frac{TN}{TN+FP}$$

## 4. Results

All the experiments were carried out over a computer with an Intel Xeon E5 Processor, 64 GB of physical memory and, the Matlab R2024a platform.

### 4.1. Image Database

For the experiments, a database consisting of 608 images of size 64×64 pixels in gray-scale pgm (Portable Gray Map) format, was used. In the database 304 images corresponds to positive stenosis cases and the remaining images are negative samples extracted from X-ray coronary angiographies. The database was previously approved by an ethics and research committee from the Mexican Institute of Social Security (UMAE-T1), under reference R-2019-1001-078, guaranteeing the privacy of the participants, prior their informed consent. The complete image database and the formed dataset will be available via web page at http://personal.cimat.mx:8181/~ivan.cruz/Journals/Stenosis608.html, accessed on 18 September 2024.

From each image, 473 features were computed, as described in [38]. There is important to mention that, for morphological features, 8 distinct vessel enhancement methods were applied: Frangi [40], Salem [41], Single and Multi-Scale Gabor filters [23,42], Multi-Scale Linear-Matched filter [22], Single and Multi-Scale Gaussian matched filters [24] and, the Top-Hat operator [25]. As result, was produced a dataset of size 608×473 rows and columns, respectively. In Table 1, are described the distinct vessel enhancement applied methods including their respective parameters value according to the state of the art.

**Figure 6:**
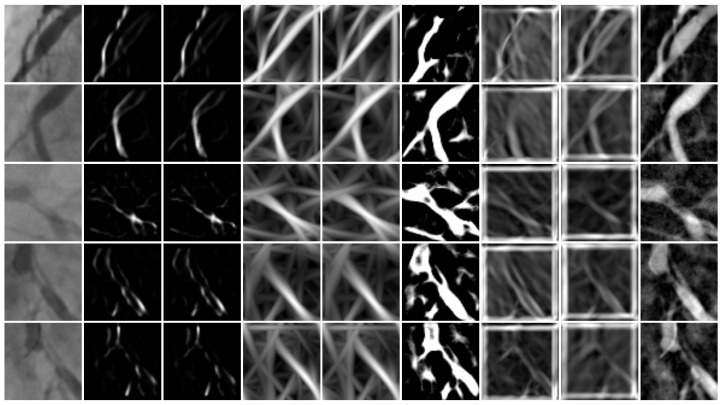
Vessel enhancement responses from 8 distinct filtering methods. Each row corresponds to a coronary patch. In columns 1 to 9, are described 8 distinct vessel enhancement methods response as follows: Original image, Frangi filter, Salem filter, Single-Scale Gabor filter, Multi-Scale Gabor filter, Multi-Scale filter, Single-Scale Gaussian-Matched Filter, Multi-Scale Gaussian-Matched Filter and, the Top-Hat operator.

Figure 6 illustrates the vessel enhancement responses corresponding to 8 distinct filtering methods for 5 angiograms, which were taken from the used image bank.

Since the image responses becoming from the distinct filtering methods are diverse, the extracted morphological features will have different values. Subsequently, the challenge is to determine which features are relevant for the classification of positive and negative coronary stenosis cases.

The dataset was partitioned into 3 groups: training, validation and testing. The training and validation groups consisted of 458 (≈75%) and 50 (≈9%) instances, respectively. The remaining 100 (≈16%) instances were used for testing purposes after the automatic feature selection stage was finished in order to prove and corroborate the classification efficiency of the proposed method.

### 4.2. Experiment Results

Using the previously formed dataset, the automatic feature selection task was performed. In this stage, a hybrid metaheuristic, involving the BUMDA and the SA methods along with a diversity control, was executed to obtain a feature subset that improves the classification performance in terms of the Accuracy metric while simultaneously decreasing the number of involved features. For the BUMDA method, the configuration included 1000 generations, a population size of 100 individuals, and a selection rate of 0.80. The SA method was set with initial and final temperature values of 1 and 0, respectively, with a constant step of 0.001, resulting in ≈1000 iterations. Since BUMDA and SA methods involve multi-objective optimization, a weight of 0.90 was assigned to classification efficiency, and 0.10 to feature reduction. In order to validate the obtained results, the proposed method was executed on 30 independent trials. All values were established as a balance between achieving correct results and minimizing the time required. Additionally, it is important to mention that, until the automatic feature selection stage, a linear SVM was used as classifier by considering the required time to produce a trained model and the number of involved individuals and generations in the distinct search metaheuristics. Subsequently, when the automatic feature selection was completed, an SVM-based classifier with fine-tuned parameters was employed. These parameters included a polynomial order, kernel scale and kernel offset with values of 5, 2 and 0.3569, respectively, as recommended in [38].

**Figure 7:**
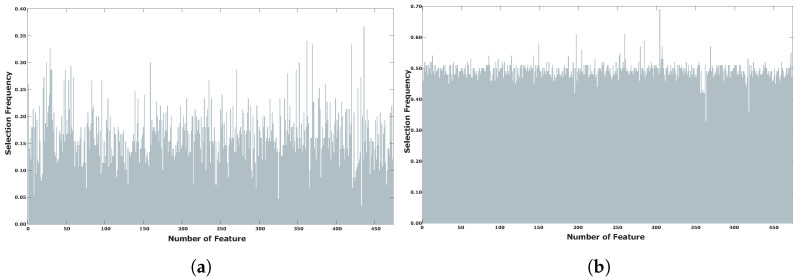
Comparison of feature selection frequency in BUMDA method (**a**) and, diversity control strategy (**b**).

In the best-achieved result, only 16 of the original 473 features were selected to classify positive and negative coronary stenosis cases with an Accuracy of 0.92 using the test dataset. Figure 7 illustrates a comparison of the feature selection frequencies for the BUMDA method and the BUMDA with diversity control applied. The frequencies were computed considering the iterations of the best trial for the traditional BUMDA and the BUMDA with explicit control diversity.

As illustrated in Figure 7, the feature selection frequencies in BUMDA with control diversity, trends to be uniform in comparison with the traditional BUMDA. This ensures a wide exploration of the search space and decreases the risk to fall into local-optima traps which is a common issue when working with metaheuristics along with high-dimensional search spaces. Table 2 presents a statistical analysis of the feature selection frequencies for the hybrid evolutionary method, comparing scenarios with and without the implementation of a population diversity strategy. Additionally, to understand the influence of considering the worst elements on population diversity, a statistical analysis of the feature selection frequency was computed over the trial with the best fitness performance.

Based on the data described in Table 2, the population diversity strategy ensures uniform feature selection frequencies in comparison with traditional approaches. The minimum and maximum values for the feature selection frequencies increased using the population diversity strategy. Subsequently, the variance and standard deviation have the lowest variations providing evidence about the uniform exploration of the search space when compared with the hybrid metaheuristic.

In addition, the use of the SA method was also relevant to improve the best and worst individual produced by the BUMDA. The best solution found by the proposed method, was the worst individual of the BUMDA population in the 730th generation. Originally, the individual consisted of 221 features and a training classification performance of 0.70, in terms of the Accuracy metric. After the SA refinement process, the number of selected features decreased to 16 and the training classification performance increased to 0.97 in terms of the Accuracy metric.

**Figure 8:**
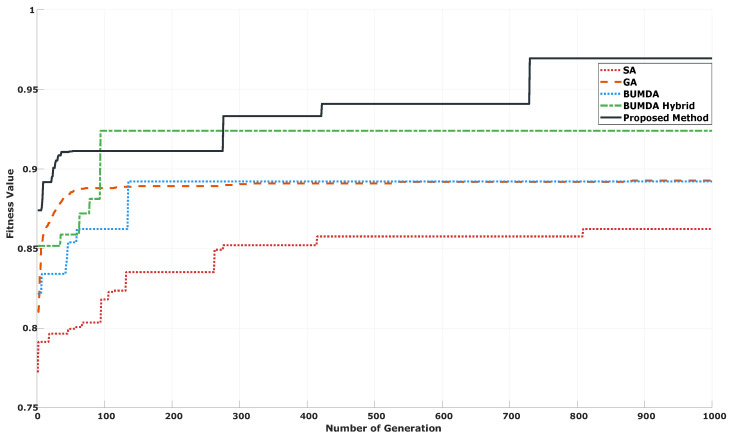
Iteration-fitness performance for the proposed method against 4 distinct methods from the literature.

An additional effect of the diversity control, is related with delaying the maximum number of generations in which the best result is achieved. In Figure 8, is illustrated the fitness performance along 1000 generations for the proposed method and contrasted with other 4 techniques taken from the literature which were adapted to the studied problem in [17].

As illustrated in Figure 8, by using a control diversity, the probability of risk for a premature convergence or stagnation is reduced considerably in comparison with traditional techniques in which only the best individuals are considered to produce new populations across the successive generations. When only the best individuals are considered to produce new solutions, the method is biased to achieve a fast convergence. In consequence, it is probably that a relevant number of potential solutions are not explored because of the discrimination of regions in the search space. However, in combinatory optimization problems, such as the automatic feature selection, there is not warranty that relevant solutions are not located close to the worst ones. By consequence, when the worst individuals are considered to produce new potential solutions, more locations into the search are explored, delaying the solution convergence. In Table 3, is described a statistical analysis of the iterations in which the best result was achieved for each method considering 30 independent trials for each one of them.

Based on the results in Table 3, the proposed method, utilizing BUMDA and SA with a population diversity strategy, tends to achieve the best results in later generations compared to other techniques. By delaying early convergence, the probability to explore the search space more exhaustively is increased. At the same time, better results can be achieved by exploring solutions close to low-fitness locations since the feature selection problem can be expressed as a combinatory-optimization computational problem in which, each variable is considered as independent from the rest of them. Notably, the optimal solution using the proposed method was found at generation number 730. In consequence, the difference between this generation number and the median and average generation numberes of the proposed method was 257 and 164, respectively, which is lower than the standard deviation of 288. This characteristic highlights the robustness of the method.

Table 4 presents a comparison of the classification performance of the proposed strategy with other state-of-the-art methods. The results were obtained using the test subset following the automatic feature selection stage.

According to the results described in Table 4, the best classification performance, in terms of the Accuracy, Jaccard coefficient, F1-score and Sensitivity, was achieved by the proposed method. On the other hand, the Hybrid Metaheuristic achieved the highest Feature Decreasing Rate and Specificity results. By comparing the results, the Hybrid-Metaheuristic trends to discriminate more features penalizing the classification performance and, according with Figure 8, the best result was achieved early during the first 100 iterations. In contrast, the proposed method was able to achieve the highest classification rate in terms of almost all metrics, in later iterations. There is important to mention that, for the deep learning-based methods, a previous transfer learning step was performed using the imagenet dataset according with [8]. However, the achieved classification performance was lower than the proposed method. This behavior could be related with an insufficient amount of images to perform a correct classification by using convolutional neural networks.

On the other hand, the achieved results using an SVM classifier showed the impact of distinct feature subsets on classification performance. Since the search space involves high-dimensional complexity, the search metaheuristics tend to converge prematurely, and the exploration occurs near possible premature local-optima locations. For this reason, the use of a strategy focused on maintaining diversity within the population of potential solutions was relevant. As illustrated previously, the solutions space is explored widely compared with the other methods. It was observed that solely selecting the best individuals from a population to produce a new generation does not guarantee the discovery of superior solutions, as exploring the contributions of less favorable individuals can yield better outcomes. As a consequence, the classification performance achieved with the proposed strategy outperformed results obtained through previous strategies. In addition, the proposed strategy achieved the highest classification performance in terms of the Accuracy, Jaccard coefficient, F1-score and Sensitivity. However, the size of the feature vector was also increased compared with the hybrid evolutionary method which not used a population diversity strategy. Table 5 details the feature vector obtained using the proposed method.

**Figure 9:**
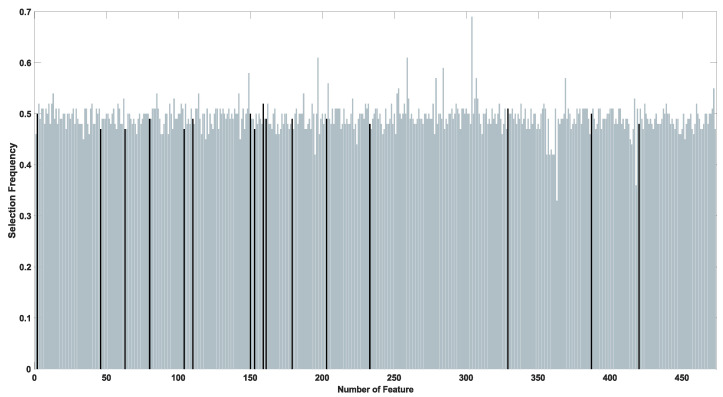
Feature selection frequencies involving the BUMDA and the SA with a diversity control, which conforms the proposed method. The previously selected 16 features are highlighted.

According to data presented in Table 5, the preprocessing methods were relevant to extract distinct morphological features. From the 8 different methods applied for vessel enhancement, 6 of them allowed the extraction of features in which the coronary stenosis classification rate was the highest. Figure 9 illustrates the selection feature frequencies related with the proposed method, highlighting the final 16 selected features.

Based on data illustrated in Figure 9 and described in Table 5, there is relevant that all texture features were discriminated by the proposed method. In contrast the formed feature subset, involves at least one feature from 7 of the 8 vessel enhancement methods that were applied. None of the features extracted from the enhanced image, using the Multi-Scale Linear-Matched Filter, were selected. In addition, two intensity-based features were relevant: the maximum intensity extracted from the original image and the standard deviation of the intensity, computed from the response of the Multi Scale Gabor Filter. The rest of the selected features are associated with the artery morphology such as length, number of vessel segments, compactness, elongatedness and circularity ratio.

The required time for the automatic feature selection task was relevant, since the size of the search space and the complexity related with the fitness function, required a considerable computational time. The minimum, maximum, median and average time in seconds, to train the SVM with the distinct feature subsets was 0.40, 4.80, 0.47 and 0.47, respectively. Once the SVM has been trained, the average time to perform the classification of a vector consisting of 16 features was 0.0003 s.

## 5. Conclusions

This paper presented an improved method aimed at increasing the classification accuracy of coronary stenosis using Accuracy and JC metrics. The use of a population diversity strategy proved crucial in exploring a broader area of the search space compared to traditional approaches. The main challenge in the feature selection process is its high-dimensional complexity, expressed as $O(2^{n})$, with n=473. The proposed strategy considers the worst-performing individuals in certain populations, which influences feature selection frequencies. As demonstrated in the results, this approach led to a higher uniformity rate in feature selection frequencies than methods that focus solely on the best individuals to produce new generations. The results validated the effectiveness of the proposed method, achieving a classification rate of 0.92 in terms of Accuracy, 0.85 for the Jaccard Coefficient, 0.92 for the F1-score, and 0.88 for Sensitivity, using only a 16 feature vector instead of the original vector of 473 features. The main strength of the proposed method lies in its ability to maintain a high classification rate based on a subset of explainable features. In contrast, deep learning methods act as black boxes and require a considerable number of instances to be trained correctly in comparison with machine learning techniques. In terms of scalability, it is important to mention that the proposed method is specialized in the classification of coronary stenosis cases. In consequence, all the morphological features are extracted from the response of image processing methods, focused on the enhancement of vessel/arterial structures. Based on this premise, the proposed method can be scaled to problems involving tubular or vessel-like structures. Finally, by considering the results described previously, involving also the required computation time to perform the classification of a single instance, the identified vector consisting of 16 features can be used in clinical practice as part of decision support information systems.

## Figures and Tables

**Table 1:** Description of the vessel enhancement applied methods involving their corresponding parameters value.

Method	Parameters	Value
Frangi, Salem	σ	[1, 12]
	$\sigma_{step}$	0.5
	α	0.5
	β	15
Single-Scale Gabor Filter	K	45
	T	5
	L	2.5
Multi-Scale Gabor Filter	I	3
	T	[2, 20]
	K	45
Multi-Scale Linear Matched-Filter	L	[1, 15]
	K	12
Single-Scale Gaussian Matched-Filter	L	13
	T	15
	σ	2.82
Multi-Scale Gaussian Matched-Filter	I	13
	T	15
	K	12
	σ	[1.5, 2.5]
	$\sigma_{step}$	0.5
Eiho Top-Hat Operator	shape	disk
	size	19

**Table 2:** Statistical analysis of the feature selection frequencies, based on the results obtained by 4 distinct search metaheuristics from the literature and the proposed method.

Method	Min	Max	Median	Avg.	Variance	Std. Dev.
GA	0.0000	1.0000	0.4010	0.4103	0.0704	0.2653
BUMDA	0.0000	0.6000	0.0333	0.0858	0.0173	0.1316
SA	0.0000	0.4200	0.0800	0.0794	0.0021	0.0455
Hybrid Metaheuristic	0.0333	0.3667	0.1600	0.1664	0.0024	0.0490
Proposed Method	0.1895	0.4830	0.3125	0.3128	0.0006	0.0242

**Table 3:** Statistical analysis related with the number of generations required for each method to achieve their best result in 30 independent trials. The proposed method was compared with 4 distinct techniques from the literature.

Method	Min	Max	Median	Avg.	Variance	Std. Dev.
SA	43	809	121	202	46,074	215
GA	79	882	126	229	52,245	229
BUMDA	97	321	188	183	4180	65
Hybrid Metaheuristic	79	203	108	117	1210	35
Proposed Method	151	998	473	566	83,245	288

**Table 4:** Comparison of classification performance and selected features considering 4 distinct methods from the literature. NSF describes the number of selected features. FDR is the feature decreasing rate. JC is the Jaccard coefficient metric. F1, Sens. and Spec., refer to the F1-score, Sensitivity and Specificity metrics, respectively.

Method	NSF	FDR	Accuracy	JC	F1	Sens.	Spec.
ResNet50 [43]	–	–	0.81	0.68	0.80	0.78	0.84
Inception-v3 [44]	–	–	0.72	0.56	0.70	0.66	0.78
VGG16 [45]	–	–	0.84	0.72	0.82	0.74	0.94
CNN-16C [7]	–	–	0.86	0.74	0.84	0.76	0.94
–	473	0.00	0.78	0.64	0.77	0.72	0.84
SA	16	0.97	0.78	0.64	0.78	0.76	0.80
GA	29	0.94	0.80	0.67	0.80	0.80	0.80
BUMDA	205	0.57	0.80	0.67	0.77	0.66	0.94
Hybrid Metaheuristic	**4**	**0.99**	0.86	0.75	0.84	0.74	**0.98**
Proposed Method	16	0.97	**0.92**	**0.85**	**0.92**	**0.88**	0.96

Best performances are remarked in bold.

**Table 5:** Description of the 16-selected features, including their corresponding type and the vessel enhancement method (VEM) used for extracting them.

Identifier	Name	Type	VEM
F002	Maximum Intensity	Intensity	-
F046	Mean Vessel Length	Morphological	Frangi
F063	Minimum Compactness	Morphological	Frangi
F080	Median Elongatedness	Morphological	Frangi
F104	Gray Level Coefficient of Variation	Morphological	Salem
F110	Median Standard Deviation of Segments Length in all Arterial Sections	Morphological	Salem
F150	Number Of Vessel Segments	Morphological	SSGF
F153	Minimum Vessel Length	Morphological	SSGF
F159	Gray Level Coefficient of Variation	Morphological	SSGF
F161	Gradient Coefficient of Variation	Morphological	SSGF
F179	Maximum Circularity Ratio	Morphological	SSGF
F203	Standard Deviation of the Intensities	Intensity	MSGF
F233	Minimum Circularity Ratio	Morphological	MSGF
F329	Maximum Standard Deviation of the Standard Deviations of Segments Length in all Arterial Sections	Morphological	MSGMF
F387	Standard Deviation of the Standard Deviations of Segments Length in all Arterial Sections	Morphological	SSGMF
F420	Max Intensity	Morphological	Top-Hat Operator

SSGF: Single-Scale Gabor Filter; MSGF: Multi-Scale Gabor Filter; MSGMF: Multi-Scale Gaussian-Matched Filter; SSGMF: Single-Scale Gaussian-Matched Filter.

## Data Availability

Data available in a publicly accessible repository http://personal.cimat.mx:8181/~ivan.cruz/Journals/Stenosis608.html accessed on 17 September 2024.

## Abbreviations

The following abbreviations are used in this manuscript:

BUMDA	Boltzmann Univariate Marginal Distribution Algorithm
FDR	Feature Decreasing Rate
GA	Genetic Algorithm
JC	Jaccard Coefficient
MSGF	Multi-Scale Gabor Filter
MSGMF	Multi-Scale Gaussian-Matched Filter
NSF	Number of Selected Features
SA	Simulated Annealing
SSGF	Single-Scale Gabor Filter
SSGMF	Single-Scale Gaussian-Matched Filter
SVM	Support Vector Machine
UMDA	Univariate Marginal Distribution Algorithm
